# A facile nanopattern modification of silk fibroin electrospun scaffold and the corresponding impact on cell proliferation and osteogenesis

**DOI:** 10.1093/rb/rbae117

**Published:** 2024-10-01

**Authors:** Xiaojiao Liu, Qinjun Ouyang, Xiang Yao, Yaopeng Zhang

**Affiliations:** State Key Laboratory for Modification of Chemical Fibers and Polymer Materials, College of Materials Science and Engineering, Shanghai Engineering Research Center of Nano-Biomaterials and Regenerative Medicine, Donghua University, Shanghai 201620, P. R. China; State Key Laboratory for Modification of Chemical Fibers and Polymer Materials, College of Materials Science and Engineering, Shanghai Engineering Research Center of Nano-Biomaterials and Regenerative Medicine, Donghua University, Shanghai 201620, P. R. China; State Key Laboratory for Modification of Chemical Fibers and Polymer Materials, College of Materials Science and Engineering, Shanghai Engineering Research Center of Nano-Biomaterials and Regenerative Medicine, Donghua University, Shanghai 201620, P. R. China; State Key Laboratory for Modification of Chemical Fibers and Polymer Materials, College of Materials Science and Engineering, Shanghai Engineering Research Center of Nano-Biomaterials and Regenerative Medicine, Donghua University, Shanghai 201620, P. R. China

**Keywords:** silk fibroin, nanopattern modification, electrospinning, cell–material interaction, plasma etching

## Abstract

As a well-known natural protein biomaterial, silk fibroin (SF) has shown broad application prospects in typical biomedical fields. However, the mostly used SF from *Bombyx mori* silkworm lacks specific cell adhesion sites and other bioactive peptide sequences, and there is still significant room for further improvement of their biological functions. Therefore, it is crucial to develop a facile and effective modification strategy for this widely researched biomaterial. In this study, the SF electrospun scaffold has been chosen as a typical SF biomaterial, and air plasma etching has been adopted as a facile nanopattern modification strategy to promote its biological functions. Results demonstrated that the plasma etching could feasibly and effectively create nano-island-like patterns on the complex surface of SF scaffolds, and the detailed nanopattern features could be easily regulated by adjusting the etching time. In addition, the mesenchymal stem cell responses have illustrated that the nanopattern modification could significantly regulate corresponding cell behaviors. Compared with the non-etched scaffold, the 10 min-etched scaffolds (10E scaffold) significantly promoted stem cell proliferation and osteogenic differentiation. Moreover, 10E scaffold has also been confirmed to effectively accelerate vascularization and ectopic osteogenesis *in vivo* using a rat subcutaneous implantation model. However, the mentioned promoting effects would be weakened or even counteracted with the increase of etching time. In conclusion, this facile modification strategy demonstrated great application potential for promoting cell proliferation and differentiation. Thus, it provided useful guidance to develop excellent SF-based scaffolds suitable for bone and other tissue engineering.

## Graphical Abstract

**Figure:**
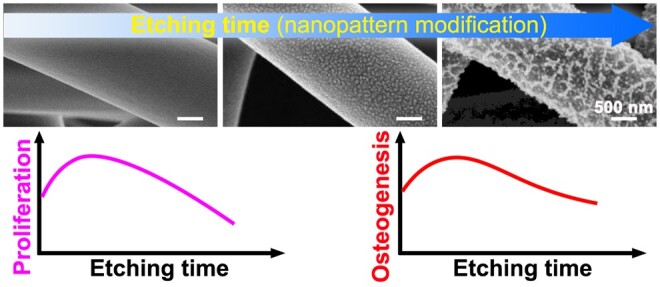

## Introduction

In typical biomedical application scenarios, suitable biomedical materials are the key factors for determining the corresponding repair and regeneration effects [1–7]. As a natural protein material, silk fibroin (SF) not only has excellent biocompatibility and adjustable biodegradability but also has the advantages of abundant sources and feasible processing ability, thus achieving wide research and applications in the typical biomedical fields [8–14].

It is well-established that SF can be processed into microspheres, fibers, membranes, electrospun scaffolds, casting scaffolds, hydrogels and other material formats for biomedical applications [10, 13]. However, the mostly used SF from *Bombyx mori* silkworm lacks specific cell adhesion sites and other bioactive peptide sequences [13]. For example, Acharya *et al*. [13, 15] have reported that fibroblasts presented better adhesion and growth on the nonmulberry SF membranes than the *Bombyx mori* SF membranes. This was attributed to the specific arginine-glycine-aspartate (RGD) sequence in the molecular chain of nonmulberry SF, which was absent in the *B.mori* SF [13, 15]. In combination with the actual performance of SF materials in the research fields of cell culture and tissue repair [16, 17], there is still significant room for further optimization of the biological function of SF materials, such as promoting initial cell adhesion, enhancing cell proliferation and guiding specific lineage commitment of stem cells. Until now, strategies based on blending bioactive components or grafting bioactive molecules have been commonly investigated and adopted to improve the cytocompatibility and biological function of SF-based biomaterials [16, 18–20]. However, for further large-scale production and application, these reported strategies confronted difficulties such as complex processes and components, high raw material costs, harsh storage and transportation conditions. To comprehensively balance the effectiveness and cost issues of the desired product application, it is quite useful and valuable to develop a facile and effective surface modification strategy for this type of widely studied SF biomaterials. Therefore, it is quite useful and valuable to develop a facile and effective modification strategy for this type of widely studied natural biomaterial, especially for its further mass production.

For the surface modification of other biomaterials, classical literature has illustrated that appropriate nanopattern modification could intensively promote cell adhesion, proliferation, migration and even directional cell differentiation [21–26]. However, the majority of the related reports have adopted patterning technologies with expensive equipment and indeed slow processing speed, such as electron beam etching, nanoimprinting and block copolymer micelle nanolithography [21, 26–29]. For example, Ruiz *et al.* [30] have reported that using electron beam etching to fabricate a template with a 95-mm patterned media disk at 1 terabit per square inch (Tb/in^2^) would take more than 1 month. Besides, most of them are just applicable for two-dimensional (2D) surface modification. In terms of further promotion and application, these nanopattern techniques still confronted difficulties such as high cost, low production efficiency and narrow material form range. Therefore, exploring an effective nanopattern modification strategy with low cost, applicable to various forms of materials, and capable of large-scale production properties holds great promise for biomedical applications.

**Figure 1.**
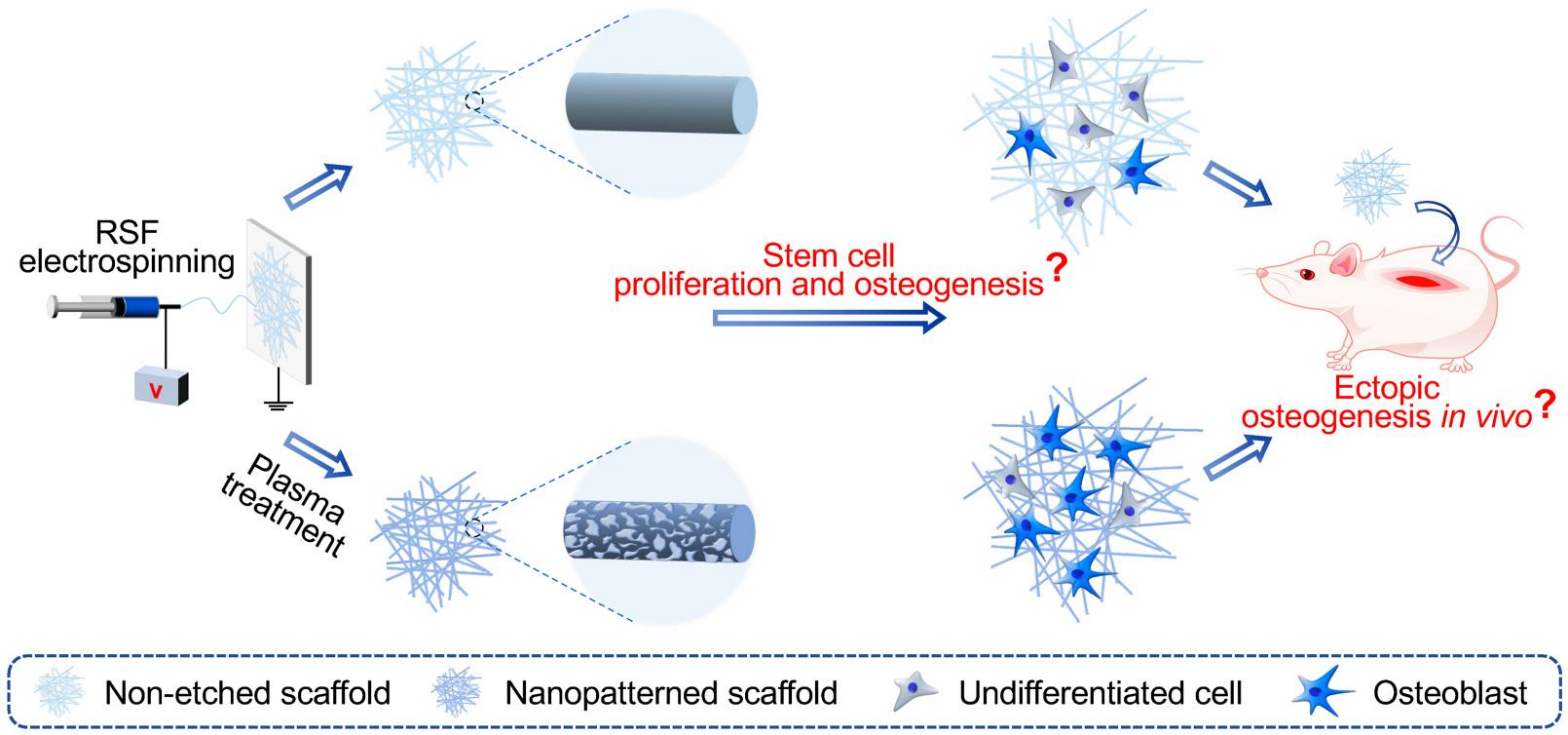
Schematic illustration of fabricating nanopatterned SF electrospun scaffold and revealing the corresponding modification effects on the proliferation and osteogenesis of stem cells *in vitro* and the ectopic osteogenic ability *in vivo*.

In this study, the SF electrospun scaffold with excellent biocompatibility and extracellular matrix (ECM) biomimetic properties was selected as a model material [5, 31–34]. The aqueous solution system for electrospinning compared to the traditional organic solvent system has unique superiorities in the field of biomedical applications, especially for the composite of growth factors and other bioactive peptides [35, 36]. Herein, a safe and non-toxic aqueous SF system was chosen to fabricate the corresponding SF electrospinning scaffold. Then, the air plasma etching technology, which has the advantages of simple operation, fast processing speed, and easy large-scale production properties, was chosen to carry out effective nanopattern modification on the irregular surface of the corresponding nanofiber scaffolds. Furthermore, considering its application potential in bone defect repair and regeneration [37, 38], the effects of nanopattern modification of SF electrospun scaffold on the proliferation and osteogenesis of bone marrow mesenchymal stem cells (BMSCs) were comprehensively evaluated. Moreover, the nanopattern modification effects on the ectopic osteogenic capacity *in vivo* have also been explored, as schematically shown in Figure 1. Air plasma etching strategy has the comprehensive superiorities of being cost-effective and convenient for large-scale production [39]. It can be used as a nanopattern modification technology with great industrialization prospects to enhance the cytocompatibility and biological functions of SF biomaterials [40]. The explorations of feasible and effective nanopattern modification strategies and the corresponding cell–material interactions could undoubtedly provide valuable inspiration for the design and development of efficient SF-based biomaterials.

## Materials and methods

### Materials

Cocoons of *B.mori* were obtained from Tongxiang, China. Regenerated cellulose dialysis bags (molecular weight cut off = 14 000 ± 2000 Da) and TRIzol were available at Shanghai Yuanju Bio-tech Co. Ltd, China. Lithium bromide (LiBr) was available at Shanghai China Lithium Industry Co., Ltd China. Na_2_CO_3_, ethanol, 4% paraformaldehyde, tertiary butanol, 1-butanol, xylene, bovine serum albumin (BSA), hydrochloric acid, aqueous ammonia, neutral balsam, trichloromethane and isopropyl alcohol were available at Sinopharm Chemical Reagent Co., Ltd China. Phosphate buffer solution (PBS), alpha-minimum essential medium (*α*-MEM), 0.25% trypsin-ethylene diamine tetraacetic acid (EDTA), fetal bovine serum (FBS) and penicillin-streptomycin were available at Gibco, USA. Tissue transparent dewaxing solution and hematoxylin-eosin (H&E) stain kit were available at Wuhan Servicebio Technology Co., Ltd China. Cell counting kit-8 (CCK-8) was available at MCE, USA. Sodium citrate antigen retrieval buffer was available at Shanghai Qianya Bio-tech Co., Ltd, China. Rabbit serum, RNA loading buffer and diaminobenzidine substrate kit were available at Beijing Solarbio Science & Technology Co., Ltd China. Tris acetate-EDTA buffer, agarose with low electroendosmosis, and diethylpyrocarbonate-treated water were purchased from Shanghai Beyotime Bio-tech Co., Ltd China. BMSCs were available at Procell Life Science & Technology Co., Ltd China. Goat anti-rabbit IgG (H + L) labeled with horseradish peroxidase was available at Abcam, UK. Green qPCR superMix, DNA marker and first-strand cDNA synthesis superMix were obtained from TransGen Bio-tech Co., Ltd China.

### Preparation of SF aqueous solution

The detailed preparation process can be found in our previous report [41]. In short, the cocoons were firstly boiled with 0.5 wt% Na_2_CO_3_ solution and washed in deionized water to obtain the degummed silk. The SF aqueous solution obtained by dissolving in 9.3 M LiBr solution was then diluted and filtered to remove impurities. Subsequently, concentrate the pure SF solution to a desired high concentration. During the following dialysis and concentration process, to prevent material denaturation or gel-forming of the SF aqueous solution, it should be carried out at a low-temperature environment of 4–8°C. More specifically, the SF aqueous solution placed into the cellulose dialysis bag was dialyzed in deionized water to remove lithium salt ions in the corresponding solution and then concentrated to approximately 15 wt% by cool airflow. Ultimately, the prepared SF aqueous solution (15 wt%) was poured into a beaker and placed on a shaker (HS 260 basic, IKA, Germany) at 120 rpm to further concentrate to about 33 wt% under cool airflow in the low-temperature environment.

### Fabrication of SF electrospun scaffolds

In this study, a metal plate with a layer of aluminum foil was selected as the collector during electrospinning. The spinning dope was SF aqueous solution with 33 wt% concentration, which was pushed by a syringe pump through a metal needle with an inner diameter of 0.6 mm. The solution propulsion speed was 1.2 ml/h. The spinning voltage and distance were set as 20 kV and 20 cm, respectively. The ambient temperature was 20 ± 5°C and the relative humidity (RH) was 45 ± 5%. After spinning, the as-spun SF scaffold was firstly dried using silica gel desiccant, then post-treated at 90% RH and 37°C for 36 h. After that, the water-insoluble SF electrospun scaffold was obtained.

### Nanopattern modification of SF electrospun scaffolds

The prepared SF electrospun scaffolds were etched with air plasma to realize different nanopattern features on their complex three-dimensional (3D) surface. The vacuum degree was fixed at 30 Pa to ensure proper operation of the plasma instrument (SY-DT01, Suzhou OPS Plasma Technology Co., Ltd, China) and a moderate degree of ionization. The etching power was fixed at half of the maximum power (150 W) of the equipment, and the etching time was modulated to regulate the nanopattern modification. As the etching time of less than or equal to 5 min could not create visible nanopattern features on the electrospun scaffold surface (see Supplementary Figure S1), herein, 10, 30 and 50 min were adopted as the etching time to create obvious nanopattern modification. The corresponding scaffolds were named the 10 min-etched scaffolds (10E scaffold), 30 min-etched scaffold (30E scaffold) and 50 min-etched scaffold (50E scaffold). The scaffold with non-etching was named the non-etched scaffold (NE scaffold).

To compare and obtain more useful information about the nanopattern features, especially the depth of the related nanopatterns, nanopatterned SF films were fabricated using the same plasma etching in the previous paragraph. The fabricated films were named NE film, 10E film, 40E film and 50E film, respectively.

### Morphology and corresponding nanopattern features observation of the electrospun scaffolds

All SF electrospun scaffolds were first sprayed with platinum with a sputtering current of 10 mA for 50 s. Subsequently, scanning electron microscopy (SEM, S-4800, Hitachi, Japan) was employed to examine the morphology and nanopattern features.

According to typical SEM images, the fiber diameter and scaffold porosity were measured and counted by Image J software. When measuring the fiber diameter in the related scaffold, the SEM images were first imported into Image J software and the corresponding scale was set. Then the related fiber diameters were manually measured by performing the ‘straight line-analyze-measure’ operations, and at least 50 individual fibers of each scaffold were measured and joined the corresponding statistics. When counting the scaffold porosity, the fiber-occupied area ratio (the ratio of the surface layer fibers project area to the corresponding SEM image area) was first counted [42, 43]. Specifically, the non-surface layer fiber regions in the SEM image were first masked with Photoshop software, and then the corresponding images were imported into Image J software. Subsequently, the fiber-occupied area ratio was obtained by performing the ‘image-adjust-threshold’ operations. Finally, the porosity of the scaffold was calculated by 100% minus this ratio according to the reported literature [42, 43]. In addition, the specifical nanopattern (nano island) features, such as the island density, island area fraction and island spacing were also carefully measured and counted from the typical SEM images using Image J software. The fiber diameter was approximately 1 μm in the electrospun scaffolds. Considering the certain curvature of the fiber surface, only the middle areas (approximately ± 0.2 μm along the central axis of the fiber) of the fibers were selected to measure and count the specifical nanopattern features.

Additionally, the nanopattern features on the etched SF films were also observed and measured. The sample preparation and SEM observation strategy was quite similar to that of the SF electrospun scaffolds. Furthermore, the atomic force microscope (AFM) (Dimension Icon, Bruker, USA) was additionally applied for recording and measuring the depth of the related nanopatterns (nano islands). Each SF film was tested in the tap mode of the AFM with a scanning range of 2.5 × 2.5 μm^2^ and a scanning speed of 1 Hz.

### Mechanical properties evaluation of the electrospun scaffolds

The scaffolds’ tensile mechanical properties were tested by a universal material testing machine (5969, Instron, USA) at 20 ± 5°C and 50 ± 5% RH. All samples were cropped into a rectangular shape (35 mm × 5 mm), and the sample thickness was measured by thickness gauge. In the mechanical properties testing process, the clamping distance was 20 mm and the tensile rate was 3 mm/min. Seven parallel samples (*n *=* *7) were set up in each group.

### Wettability evaluation of the electrospun scaffolds

The contact angles of the scaffolds were obtained with a water contact angle (WCA) goniometer (OCA40 Micro, Dataphysics, Germany) to evaluate their wettability. All the images used for measuring the corresponding contact angles were taken 2 s after the water droplets contacted the surface of the scaffold.

### Cell culturing

BMSCs of passage 0 to passage 1(P0 ∼ P1) were cultured in the cell culture medium (*α*-MEM medium:FBS:penicillin-streptomycin solution = 100:10:1) and grown at 37°C and 5% CO_2_ atmosphere. When approximately 85% of the culture flask bottom was covered with cells, it was necessary to digest cells for a subculture to obtain a sufficient number of cells for subsequent experiments. As for the cell proliferation and differentiation evaluation experiments, only the cells less than P5 (passage 5) were used.

### Cell seeding and culturing

Cut the SF electrospun scaffolds to fit the size of the 24-well plate and fix them to the bottom of the plate with customized stainless-steel rings. Subsequently, the immobilized scaffolds were immersed in 75 vol% sterile ethanol for 2 h and rinsed thoroughly with PBS. Then, the BMSCs were seeded onto the scaffolds with a density of 4 × 10^4^ cells per well, and the volume of the culture medium was 500 μl per well. After 6 h of culture, the suspended cells in each well were removed by removing all the culture media and immediately replaced with a fresh culture medium. Then, it was replaced every 2 days to continue cell culture.

### Cell adhesion and proliferation evaluation

After culturing for 1 or 4 days, the cell adhesion situation on each scaffold was observed by SEM. Before observation, the material-cell mixture was firstly immersed in 4 vol% paraformaldehyde in PBS for 3 h at 4°C, and then subjected to gradient dehydration in a series of 30, 50, 70, 75, 80, 90, and 100 vol% ethanol solution successively, soaking for 10 min under each condition. After drying, they were sputtered with platinum (10 mA for 50 s) and observed by using SEM (S-4800, Hitachi, Japan).

Cell proliferation on different scaffolds during the initial 4 days was detected by CCK-8 assay. Briefly, after culturing for 1 or 4 days, 500 μl CCK-8 working solution (*α*-MEM medium with 10% FBS and 10% CCK-8 reagent) replaced the corresponding culture medium. After incubating for 2 h, 200 μl working solution per well was aspirated for testing. After that, at a wavelength of 450 nm, the optical density (OD) value of the incubated working solution was obtained from the microplate reader (Multiskan FC, Thermo Fisher Scientific Inc, USA). Finally, the cell proliferation was evaluated by the changes in OD value from the 1st to the 4th day.

### Cell differentiation evaluation

For evaluating the influence of nanopattern modification on the stem cell osteogenic differentiation, the expression of osteogenic-related genes, such as alkaline phosphatase (ALP), osteocalcin (OCN), osteopontin (OPN) and Runt-related transcription factor 2 (Runx2) was detected by reverse transcriptase polymerase chain reaction (RT-PCR) technology.

Specifically, cell seeding was performed as the protocol in section ‘Cell seeding and culturing’. After culturing for 7 days, TRIzol was used for lysing cells. Trichloromethane was added to the lysate, and the upper aqueous phase containing total RNA was obtained after centrifugation. Isopropyl alcohol was added further and the RNA precipitate was obtained after centrifugation. Finally, diethylpyrocarbonate-treated water was added to dissolve the RNA precipitate to send samples for RT-PCR testing. The RNA reverse transcription was achieved with first-strand cDNA synthesis superMix. The RNA reverse transcription and following fluorescence quantitative detection were performed by Shanghai Qianya Bio-tech Co. Ltd, China. The primers of ALP, OCN, OPN, Runx2 and internal reference gene (β-actin) were listed in Table 1. The 2^-ΔΔCt^ method was used for analyzing related data.

**Table 1.** RT-PCR primer sequences of the tested genes

Primer	Forward (5′–3′)	Reverse (5′–3′)
ALP	GAAAGAGAAAGACCCCAGTTAC	ATACCATCTCCCAGGAACAT
OCN	AAAGCCCAGCGACTCTGA	CTCCAAGTCCATTGTTGAGGT
OPN	CGCATTACAGCAAACACTCAG	GTCATCGTCGTCGTCATCAT
Runx2	CGAAATGCCTCTGCTGTTAT	CGTTATGGTCAAAGTGAAACTCT
β-actin	CCTCTATGCCAACACAGT	AGCCACCAATCCACACAG

### Ectopic osteogenic ability assessment *in vivo*

To evaluate the nanopattern modification on the osteogenesis *in vivo*, a typical back subcutaneous implantation rat model was used to assess the ectopic osteogenic ability of NE scaffold, 10E scaffold, 30E scaffold and 50E scaffold. This work focused on revealing the effect of SF electrospun scaffolds with or without nanopatterned modification on the osteogenic capacity, so the negative control without scaffold implantation was not included. It has been reported that the implantation of SF electrospun scaffolds without additional treatment could significantly promote bone repair compared with the negative control without scaffold implantation in a bone-injured rabbit model [44]. All *in vivo* experiments in this research were authorized by the Animal Care and Ethics Committee of Donghua University (license for the approval of ethical review for experimental animals: DHUEC-NSFC-2023-20; license for the use of experimental animals: SYXK (Shanghai) 2020-0018).

Specifically, SD rats (160–180 g, *n *=* *5) were anesthetized by intramuscular injection using Sutex^®^ 50 at 1 ml/kg. After disinfection with iodophor, the backs of rats were incised and sterile SF electrospun scaffolds were implanted. The material-cell mixture was taken out after 14 and 21 days of implantation. Further immunohistochemical staining and RT-PCR were taken to evaluate the ectopic osteogenic abilities of the nanopatterned or non-etched SF electrospun scaffolds.

Immunohistochemical staining procedure: The material-cell mixture was firstly immersed in 4 vol% paraformaldehyde for 24 h at 20 ± 5°C. After dehydration, immersing in paraffin, embedding and sectioning (4 µm thick) were then performed. The sections were deparaffinized and washed with water. After antigen repair and serum closure, the material-cell mixture was stained with OCN monoclonal antibody (MA1-20786, Invitrogen, USA) at 4°C for 12 h. After PBS washing, they were dipped into horseradish peroxidase-labeled goat anti-rabbit IgG (H + L) (ab205718, Abcam, UK) as a secondary antibody for further staining at room temperature for 50 min. Subsequently, a diaminobenzidine substrate kit was used for labeling the fixed second antibody on the samples. The working solution concentrations of all the dye reagents are according to the recommended concentrations in the corresponding product manuals. Ultimately, the samples were observed with an optical microscope (Eclipse Ci, Nikon, Japan). The positive expression of OCN was labeled as brown.

The expression of osteogenesis-specific genes (ALP, OCN, OPN and Runx2) was detected by RT-PCR. Briefly, the material-cell mixture was minced and homogenized in TRIzol until the cells were completely lysed. The RNA reverse transcription and following RT-PCR detection were performed by Shanghai Qianya Bio-tech Co. Ltd, China. The primers of the related genes and specific RT-PCR testing process were the same as described in section ‘Cell differentiation evaluation’.

To assess the biocompatibility of the mentioned scaffolds, hematoxylin and eosin (H&E) staining was also performed to stain the material-cell mixture after implantation at 14 and 21 days. The corresponding fixation, embedding, sectioning and deparaffinization of the material-cell mixture was performed as the immunohistochemical staining process mentioned in the third paragraph of section ‘Ectopic osteogenic ability assessment *in vivo*’. After that, the nuclei were stained with hematoxylin and the cytoplasm was stained with eosin. Finally, the cells were observed and photographed with an optical microscope (Eclipse Ci, Nicon, Japan). The nuclei and cytoplasm were labeled as blue and red, respectively.

### Statistical analysis

The statistics of all data were represented by mean ± standard deviation (*n *≥3). The differences between indicated samples were assessed using one-way ANOVA analysis, with *P *<* *0.05 representing a significant difference.

## Results and discussions

### Morphological and corresponding nanopattern features of the SF electrospun scaffolds

The morphology characteristics of the electrospun scaffolds, such as the fiber features and scaffold porosity, have also been found to profoundly impact cell function regulation [33, 45–47]. In addition, it has been reported that some typical nano cues, such as nanopattern distance and nanopattern depth could effectively regulate cell behaviors [22, 25, 48–50]. Consequently, the important morphological and corresponding nanopattern features of the non-etched or plasma-etched SF electrospun scaffolds were carefully evaluated and compared.

**Figure 2.**
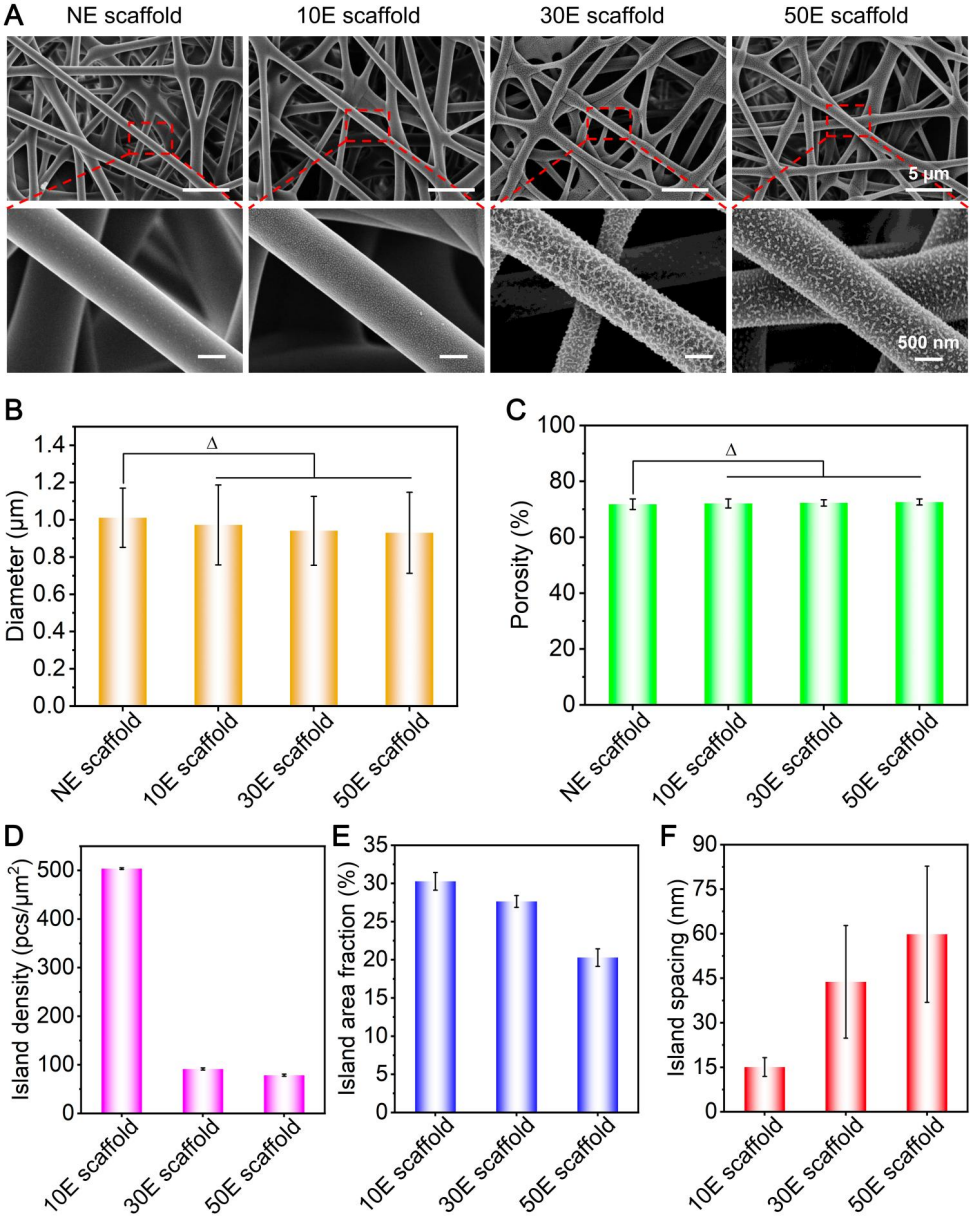
The morphological and corresponding nanopatterned features of the non-etched or plasma-etched SF electrospun scaffolds with different etching times. (**A**) SEM images, (**B**) fiber diameter, (**C**) scaffold porosity, (**D**) island density, (**E**) island area fraction and (**F**) island spacing. ‘Δ’: *P *>* *0.05.

Typical SEM images of NE scaffold, 10E scaffold, 30E scaffold and 50E scaffold were shown in Figure 2A. The possible effects of plasma etching on the fiber diameter and scaffold porosity were carefully assessed from typical SEM images. Results illustrated that the average fiber diameters of NE scaffold, 10E scaffold, 30E scaffold and 50E scaffold were 1.01 μm, 0.97 μm, 0.94 μm and 0.93 μm, respectively (Figure 2B), and the porosities of them were approximately 71.8%, 72.0%, 72.3% and 72.6%, respectively (Figure 2C). Probably because of the etched patterns within the nanoscale ranges, both fiber diameters and scaffold porosities showed no significant difference between the plasma etched or non-etched scaffolds.

To explore the effect of plasma etching time on the nanopatterns, we further analyzed the surface morphology of the prepared scaffolds. The surface of fibers in NE scaffold was extremely smooth, while those in air plasma etched scaffolds all presented obvious nano island-like patterns. Moreover, the nanopatterns could be successfully formed even in the inner layer of 10E scaffold, 30E scaffold and 50E scaffold (Figure 2A). Overall, the distance between nano islands increased with the extension of etching time. The shape of the islands gradually changed from point-like to irregular worm-like nanopatterns (Figure 2A). Furthermore, the specific features of the fabricated nanopatterns on the indicated scaffolds were measured and statistically analyzed. The average values of island densities for 10E scaffold, 30E scaffold and 50E scaffold were approximately 504 pcs/µm^2^, 91 pcs/µm^2^ and 78 pcs/µm^2^, respectively (Figure 2D), which showed a sharp decreasing tendency along with the increase of etching time. In addition, the island area fraction, that was, the ratio of the total island area to the whole surface area of the fiber, was also measured and calculated. The mean values of the island area fraction for 10E scaffold, 30E scaffold and 50E scaffold were approximately 31%, 27% and 20%, respectively (Figure 2E), which showed a gradual decreasing trend with the increase of etching time. The mean values of the island spacing were about 15 nm, 44 nm and 60 nm, respectively (Figure 2F), which showed an increasing tendency with the extension of etching time. It is noteworthy that the nanopattern features were similar to the SF film fabricated under the same etching conditions (Supplementary Figure S2). These results further proved and confirmed the universality of the air plasma etching strategy for modifying obvious nanopatterns on the surface of SF materials. Moreover, the height or depth of the fabricated nano-islands on the 2D surface (film) has been evaluated using AFM images (Supplementary Figure S3). Results showed that the island height measured using AFM software gradually increased from 13 nm to 56 nm with the increase of etching time (Supplementary Figure S3), which indirectly reflected the height or depth information of the fabricated nano-islands on the surface of the fibers in the SF electrospun scaffold.

These nano-islands were probably the more stable and hard regions (with high content of β-sheet structures) [51–53] resulting from the preparation and post-processing of the SF electrospun scaffolds, whereas the regions etched away from the fiber surface were more likely the soft and relative unstable regions (with high content of amorphous structures) [54, 55]. As the etching time became longer, the soft regions were gradually etched away from the outer toward the inner layers, and the hard regions were gradually exposed and partially fused. As the etching time increased, only the core regions of the hard part remained. Specifically, when etched for 10 min, the hard regions were gradually exposed, showing a point-like island morphology. At this condition, the island density and area fraction were the highest, while the spacing between islands was the smallest. When etched for 30 min, all the hard regions were exposed and the majority of the adjacent hard regions fused, presenting an irregular worm-like island morphology. Consequently, the island density sharply decreased, the island area fraction gradually decreased, while the island spacing gradually increased. When etched for 50 min, even parts of the mentioned ‘hard regions’ would be etched away, and only the core regions of the hard regions would remain. At this condition, the island density and area fraction were the smallest, while the island spacing was the largest.

In summary, the mentioned plasma etching strategy could feasibly create obvious nanopattern (nano-island-like) features on the 2D film and 3D scaffold surface of SF materials, and the nanopattern characteristics could be easily regulated by adjusting etching time. When the etching time is no more than 50 min, no significant difference was found for the fiber diameter and scaffold porosity of the scaffold.

### Effects of the nanopattern modification on the hydrophilicity and mechanical properties of SF electrospun scaffolds

The hydrophilicity and mechanical property are also important features that need to be considered for biomaterials. Therefore, we measured the WCA of different nanopatterned SF electrospun scaffolds and their tensile mechanical properties.

**Figure 3.**
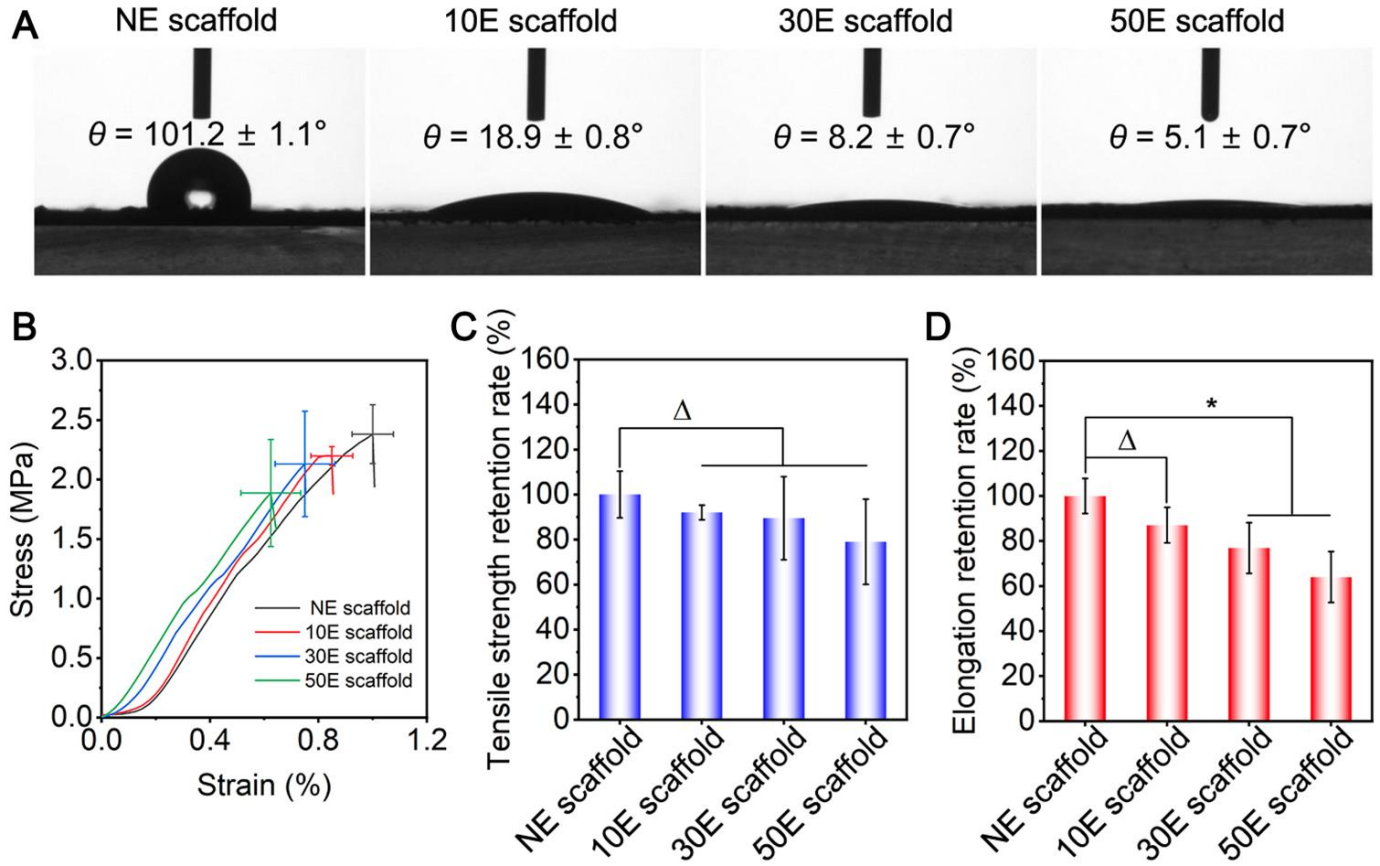
Hydrophilicity and mechanical properties of the non-etched or plasma-etched SF electrospun scaffolds with different etching times. (**A**) WCA results, (**B**) stress–strain curves, (**C**) tensile strength retention rate, (**D**) elongation retention rate. ‘Δ’: *P *>* *0.05, ‘*’: 0.01 < *P *≤* *0.05.

The WCA for NE scaffold, 10E scaffold, 30E scaffold and 50E scaffold were about 101.2°, 18.9°, 8.2° and 5.1°, respectively (Figure 3A). The hydrophilicity of these scaffolds sharply increased after air plasma etching and further increased with etching time. This was probably attributed to the significant increase in hydrophilic functional groups and roughness of the scaffold surface. For one thing, abundant oxygen-containing and nitrogen-containing functional groups such as carboxylic groups (-COOH) and hydroxyl groups (-OH) could be introduced on the surface of electrospun scaffolds after air plasma etching [56, 57], thus significantly increasing the polarity of the scaffold surface and led to a sharp increase in the hydrophilicity [56, 57]. For another thing, the nanopatterns that formed after air plasma etching improved the surface roughness [58], which increased the contact area of water molecules and made it easier for water spreading, thus further increasing the material hydrophilicity.

The mechanical strength required for stressed tissues (such as bone tissue) is relatively high, while that required for non-stressed tissues (such as skin and liver tissue) is relatively low. Some reports have illustrated that plasma etching may significantly affect the mechanical properties of polymer materials, thereby limiting their application scenarios [59, 60]. Therefore, the strain–stress curves of the SF electrospun scaffold were analyzed in this study (Figure 3B). The tensile strength of NE scaffold, 10E scaffold, 30E scaffold and 50E scaffold were approximately 2.38 MPa, 2.20 MPa, 2.13 MPa and 1.89 MPa, respectively. The elongation at the break of these scaffolds was about 0.97%, 0.85%, 0.75%, and 0.63%, respectively. Compared to NE scaffold (raw material), 10E scaffold, 30E scaffold and 50E scaffold retained 92%, 89% and 79% of the tensile strength (Figure 3C), and retained 87%, 77% and 64% of the elongation at break, respectively (Figure 3D). It indicated that after 10 min of plasma etching, the tensile strength and elongation at the break of the SF electrospun scaffold have a limited decrease (ca. 10%). When the etching time was extended, the tensile strength of the scaffold further slightly decreased (Figure 3C), while the elongation at break significantly reduced (Figure 3D). That is probably because plasma etching destroyed the structure on the surface of SF electrospun fibers to a certain extent. The relevant decreasing trend of mechanical properties was consistent with the reported effect of plasma etching on the mechanical properties of conventional polymer materials [59, 61, 62].

These results indicated that the air plasma etching could increase the hydrophilicity of the scaffold surface. In addition, the tensile strength and elongation at the break of the scaffold were decreased as the extension of etching time.

### Effects of the nanopattern modification of SF electrospun scaffolds on cell adhesion and proliferation

Good cytocompatibility is usually required for effective tissue repair [63–65]. Therefore, it is quite important to reveal the effects of different nanopatterned scaffolds on the adhesion and proliferation of cells.

**Figure 4.**
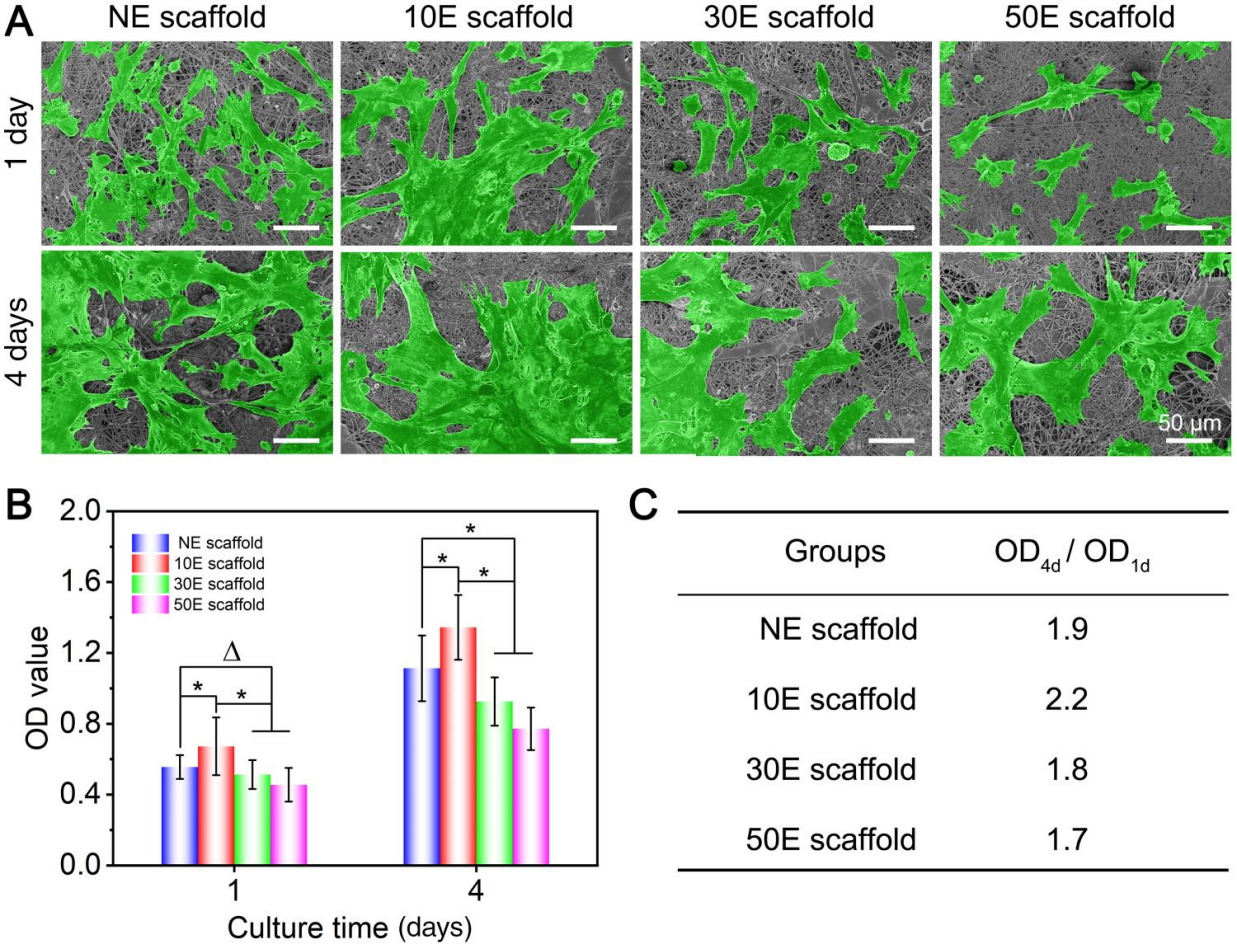
Adhesion and proliferation of stem cells on the non-etched or plasma-etched SF electrospun scaffolds with different etching times. (**A**) SEM images (the cells were pseudo-colored green), (**B**) OD value of the CCK-8 testing to reflect the total cell viability (‘Δ’: *P *>* *0.05, ‘*’: 0.01 < *P *≤* *0.05), (**C**) OD value change rates (OD_4d_/OD_1d_) of (B) to reflect the corresponding cell proliferation rate.

From the initial cell adhesion results (Figure 4A), the maximum number of cells was found on 10E scaffold, followed by NE scaffold and 30E scaffold. The smallest cell number was found on 50E scaffold. This result initially suggested that 10E scaffold was more favorable for cell adhesion. On day 4, the cell number on the surface of all electrospun scaffolds was significantly higher than those on day 1. Moreover, the cell number on 10E scaffold and NE scaffold was significantly higher than that on 30E scaffold and 50E scaffold (Figure 4A). These results qualitatively indicated that 10E scaffold and NE scaffold are more favorable for cell proliferation.

To further quantitatively characterize the cell cytocompatibility, total cell viability was assessed after 1 and 4 days of culture, respectively. After 1 day of culture, the cell viability on 10E scaffold was significantly higher than those of the other three groups (Figure 4B). After 4 days of culture, the cell viability of all four scaffolds showed a significant increase. The OD value of cells on 10E scaffold remained the highest among the four groups. The OD value change rates (OD_4d_/OD_1d_) represented the rate of cell growth. Further calculations showed that the OD value change rates of NE scaffold, 10E scaffold, 30E scaffold and 50E scaffold were 1.9, 2.2, 1.8 and 1.7, respectively (Figure 4C). These data indicate that cells on 10E scaffold grew fastest among the four scaffolds. When compared to NE scaffold, these results comprehensively revealed that 10E scaffold promoted cell adhesion and proliferation, while 50E scaffold restricted the related cell behaviors to some extent. This phenomenon could well coincide with the imaging results in Figure 4A.

The ability to promote cell adhesion and proliferation from high to low was 10E scaffold, NE scaffold, 30E scaffold and 50E scaffold, respectively. This is probably due to the comprehensive effect of the surface wettability and the nanopattern features of the electrospun scaffolds. Compared to NE scaffold, cell adhesion and proliferation were increased on 10E scaffold. The main reason probably is that the point-like nanopatterns with an average interspacing of approximately 15 nm were formed on the fiber surface of 10E scaffold. For one thing, the corresponding nanopatterns increased the roughness of the scaffold surface and provided more effective protein and cell anchoring sites. Some previous reports have illustrated that high roughness surfaces and nanodots patterned surfaces with smaller interspacing (less than 70 nm) were more appropriate for cell adhesion and proliferation [28, 66–68]. For another, 10E scaffold also presented a more suitable hydrophilic surface (WCA, ca. 19°) than the hydrophobic surface (WCA, ca. 101°) of NE scaffold. It has been reported that the hydrophobic materials were not conducive to cell adhesion because they could prevent physical contact between the cell and material surface [69, 70]. When the etching time was extended to 30 and 50 min, the WCA of the scaffolds decreased dramatically to 5–8°, which caused the corresponding scaffold surfaces to become excessively hydrophilic. Typical literature has reported that a drastic weakening of hydrophobic–hydrophobic interactions between proteins and excessive hydrophilic substrate (WCA less than 10°) in aqueous cell culture condition has reduced protein adsorption on the substrate, thus not conducive to cell adhesion [71, 72]. Additionally, as the etching time becomes longer, the spacing between nano-islands on the fiber surface has also gradually increased (15 nm–44 nm–60 nm). Wang *et al.* [50] indicated that the interspacing features of nanodot patterns also played vital roles in influencing cell adhesion. Their research revealed that as the interspacing increased from 37 to 124 nm, the cell density and individual cell spreading both showed a gradually decreasing trend [50]. It has also been reported that the air plasma etching could probably induce some reactive oxygen species on the material surfaces [73]. Excessive levels of reactive oxygen species that exceed the cell’s clearance capacity could generate some unfriendly effects on the cells and even cause cell damage [73]. These are likely to be other reasons for the weaker cell adhesion and proliferation on the longer time plasma etched scaffolds (especially 50E scaffold) when compared with 10E scaffold.

In summary, the developed nanopattern modification of SF electrospun scaffolds significantly regulates stem cell adhesion and proliferation. Compared with NE scaffold, suitable air plasma etching could significantly increase the corresponding cell behaviors, while longer plasma etching time could even restrict cell behaviors.

### Effects of the nanopattern modification of electrospun scaffolds on cell osteogenesis

As for the special application of the tissue engineering scaffold, a desired differentiation induction ability is another important cue impact on its final repair effect. Some typical studies have confirmed the feasibility of SF electrospun scaffold in the field of bone tissue repair [38, 74]. Ideal bone tissue engineering materials should have good osteoinductive ability. Therefore, the effects of nanopattern modification of electrospun scaffolds on the osteogenesis of stem cells were explored.

**Figure 5.**
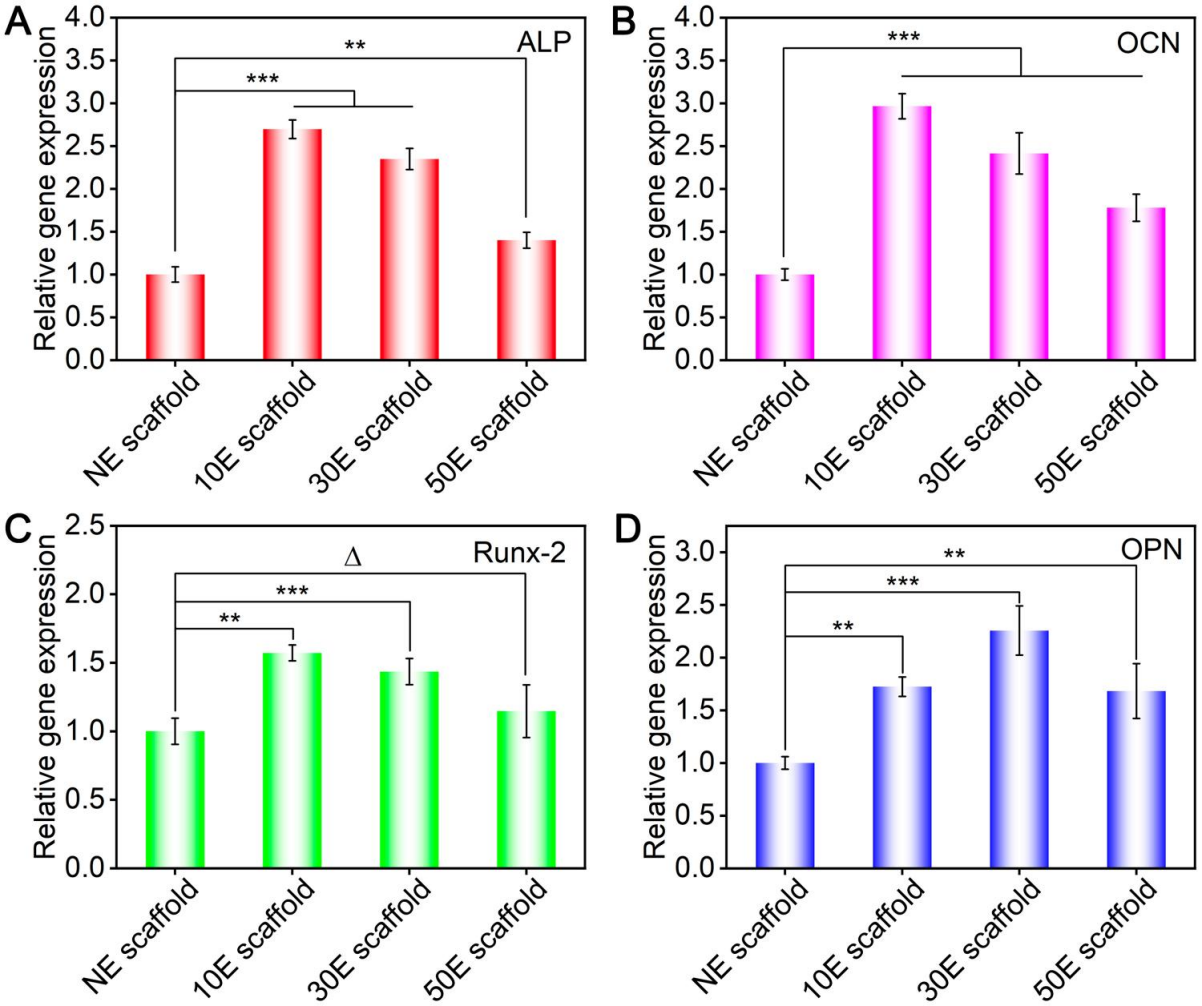
Osteogenesis-specific gene expression of BMSCs on the non-etched or plasma-etched SF electrospun scaffolds with different etching times after 7 days of culture. (**A**–**D**) Relative gene expression of ALP, OCN, Runx2 and OPN, respectively. ‘Δ’: *P *>* *0.05, ‘**’: 0.001 < *P *≤* *0.01, ‘***’: *P *≤* *0.001.

The osteogenesis-specific gene (ALP, OCN, Runx2 and OPN) expression of BMSCs after 7 days of culture on NE scaffold, 10E scaffold, 30E scaffold and 50E scaffold was shown in Figure 5. As for ALP, OCN and Runx2, the order of gene expression from high to low is 10E scaffold, 30E scaffold, 50E scaffold, and NE scaffold, respectively (Figure 5A–C). As for the expression of OPN, the order of gene expression from high to low is 30E scaffold, 10E scaffold, 50E scaffold and NE scaffold, respectively (Figure 5D). Comprehensively, the nanopatterned SF electrospun scaffolds were more conducive to stem cell osteogenic differentiation than NE scaffolds. Among these scaffolds, 10E scaffold presented the strongest ability to promote stem cell osteogenesis. As for the plasma etched scaffolds, the mentioned osteogenesis induction abilities decreased with the increase of etching time when comparing 10E scaffold, 30E scaffold and 50E scaffold.

10E scaffold showed the optimal stem cell osteogenic induction capacity. As mentioned before, the significantly increased roughness, appropriate point-like nanopatterns and their smaller interspacing features on the fiber surface of 10E scaffold are all beneficial for cell adhesion, spreading and proliferation. Larger cell spreading and higher cell density were proven to enhance stem cell osteogenesis [22, 75]. In addition, the enhanced cell adhesion capacity of single fibers would also promote cell adhesion between adjacent fibers in the electrospun scaffold. Related pioneer research has precisely revealed that the cell cross-adhesions between adjacent fibers could further enhance stem cell osteogenesis [47]. Comprehensively, the optimal osteogenesis capacity occurred on 10E scaffold.

What is more, Wang *et al*. [50] conducted a more in-depth differentiation comparative study on the surface of the non-fouling substrate and found that the interspacing feature of arginine-glycine-aspartic acid (RGD) nanodots itself was a unique independent factor affecting cell osteogenesis. Relevant studies indicated that as the increasing of the interspacing of RGD nanodots (ranging from 37 to 124 nm), the proportion of osteogenic differentiated cells increased. In this study, the interspacing of nano-islands on the surface of 30E scaffold and 50E scaffold was approximately 44 nm and 60 nm, respectively (Figure 2F). That is probably why the osteogenesis capacities of the nanopatterned scaffolds were all better than those of the non-etched scaffold (NE scaffold). As for the comparison of 30E scaffold and 50E scaffold to 10E scaffold, the cell adhesion (spreading) and cell densities were both smaller, thus presenting lower osteogenic induction ability.

### Effects of the nanopattern modification on the ectopic osteogenic abilities of SF electrospun scaffolds

**Figure 6.**
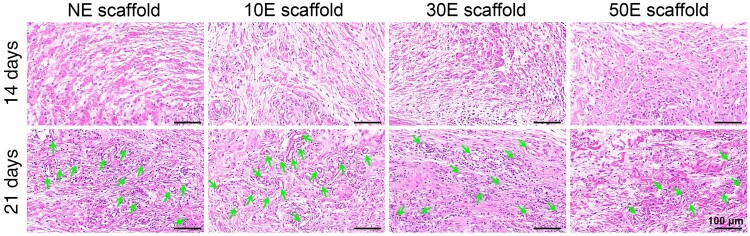
H&E staining of the indicated material-cell mixture after 14 and 21 days of implantation (inserted arrow: hollow circular ring structures).

To further evaluate the *in vivo* performance of the nanopattern-modified SF electrospun scaffolds, a rat subcutaneous implantation model in the back was adopted. After 14 and 21 days of subcutaneous implantation, the biocompatibility of different nanopatterned scaffolds was evaluated by H&E staining (Figure 6). After 2 weeks of implantation, all plasma-etched groups presented similar staining results compared to the non-etched group (NE-scaffold). Only mild inflammatory response could be found on all the SF electrospun scaffolds, which coincided with the reported high biocompatibility of SF biomaterials [8, 76, 77]. After 3 weeks of implantation, all four groups presented significantly more cell numbers, and most of them showed an obvious vascularization effect (hollow circular ring structures, Figure 6). The number of hollow circular ring (blood vessel-like) structures in the typical captured regions (see Figure 6) of NE scaffold, 10E scaffold, 30E scaffold and 50E scaffold were approximately 13, 16, 11 and 7, respectively. These results demonstrated that the mentioned nanopattern modification would not change the good biocompatibility of the SF electrospun scaffold, which was guaranteed by the useful aqueous solution electrospinning system of SF. Compared with other groups, 10E scaffold presented a better ability to promote angiogenesis (Figure 6). This may be attributed to the fact that 10E scaffold was better suited to cell adhesion and proliferation, thus more favorable for recruiting for and communicating with the surrounding cells and tissues *in vivo*.

**Figure 7.**
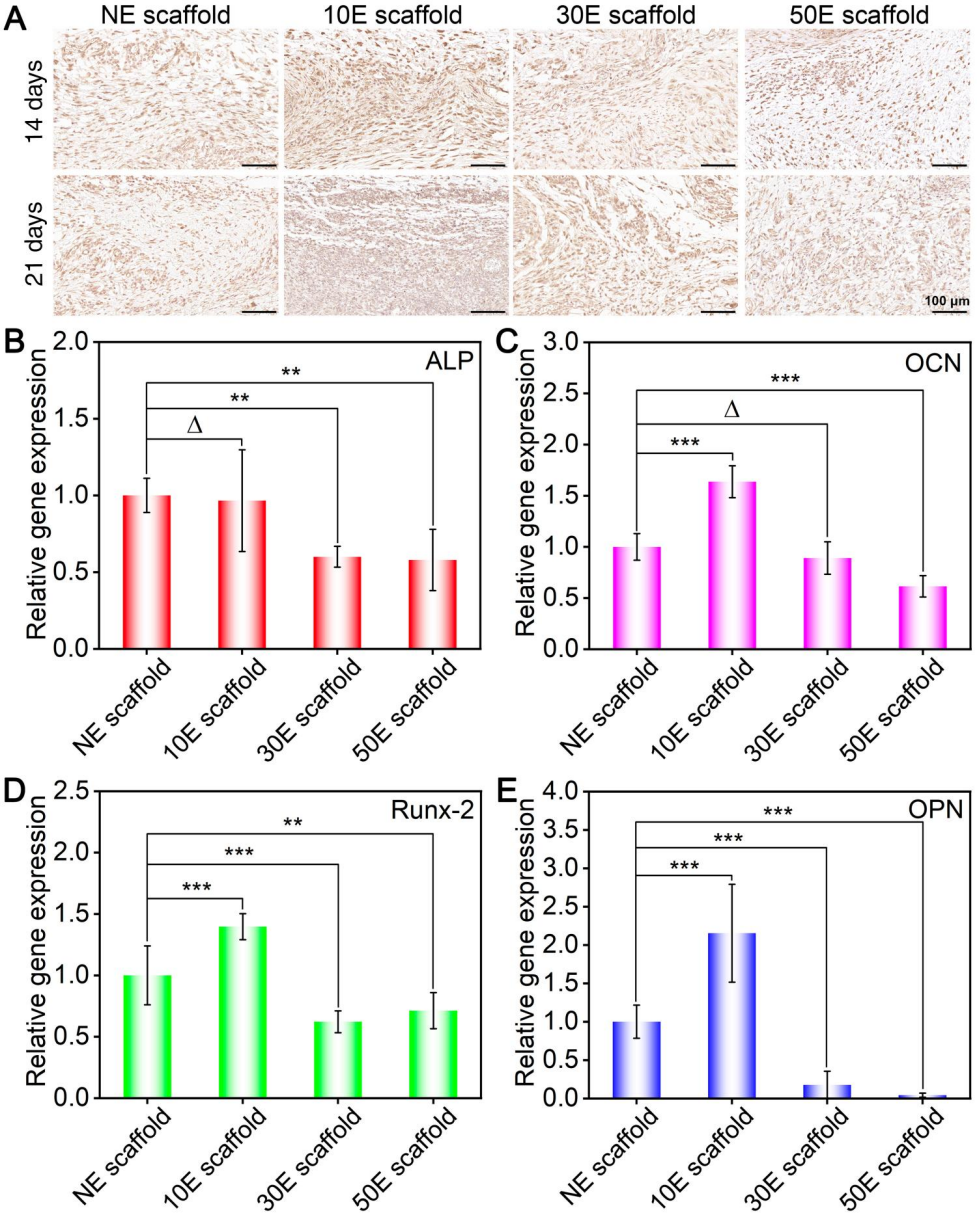
Ectopic osteogenesis of the indicated SF electrospun scaffolds implanted subcutaneously. (**A**) OCN immunohistochemical staining of the indicated material-cell mixture after 14 and 21 days of implantation, (**B**–**E**) relative genes expression of ALP, OCN, Runx2 and OPN, respectively. ‘Δ’: *P *>* *0.05, ‘**’: 0.001 < *P *≤* *0.01, ‘***’: *P *≤* *0.001.

To confirm the potential of the nanopatterned scaffold for tissue engineering, ectopic osteogenic abilities of the scaffolds were assessed in the back subcutaneous microenvironment of rats. Both immunohistochemical staining and RT-PCR technologies have been applied to this *in vivo* experiment. After 14 and 21 days of subcutaneous implantation, the OCN immunohistochemical staining results of different material-cell mixtures are shown in Figure 7A. It could be found that the expression of OCN (stained as brown) on 10E scaffold was the highest, while that on the 50E scaffold was the lowest.

After 3 weeks of implantation, the osteogenesis-specific gene (ALP, OCN, Runx2 and OPN) expression in the corresponding material-cell mixture has also been detected by RT-PCR (Figure 7B–E). Results illustrated that the highest gene expression of all four genes happened on 10E scaffold. In contrast, 50E scaffold showed the lowest specifical gene expression among these groups. These RT-PCR results can be well matched with the immunohistochemical staining trend (Figure 7A).

Comprehensively, it was found that the highest specific protein and gene expressions happened on 10E scaffold, which indicated that it owned the strongest ectopic osteogenesis capacity. In contrast, the lowest specific protein and gene expressions occurred on 50E scaffold. Both the *in vitro* and *in vivo* studies have confirmed that 10E scaffold owned the optimal osteogenic differentiation induction capacity. It needs to be noted that the lowest specific gene expression happened on NE scaffold *in vitro* (Figure 5), while the weakest osteogenesis induction ability happened on 50E scaffold *in vivo* (Figure 7). Different from the *in vitro* cell culture system, better vascularization is essential for nutrient exchange and metabolite elimination in the *in vivo* microenvironment and further promotes the corresponding osteogenesis *in vivo*. As mentioned in section ‘Effects of the nanopattern modification of SF electrospun scaffolds on cell adhesion and proliferation’, combined with the effects of scaffold surface wettability and nanopatterned features on cell adhesion and proliferation, 50E scaffold showed the worst cell adhesion and proliferation. The restricted cell adhesion and proliferation behaviors of 50E scaffold (Figure 4) probably slow down the related vascularization *in vivo* (Figure 6), thus reducing the mentioned ectopic osteogenesis (Figure 7). That is perhaps why 50E scaffold presented the lowest osteogenesis induction capacity *in vivo*. As for 10E scaffold, it could not only significantly enhance the cell proliferation (Figure 4) and osteogenesis (Figure 5) directly, but also obviously promote the related vascularization (Figure 6), thus displaying the highest osteogenesis induction capacity *in vivo* (Figure 7).

Comprehensively, both the non-etched and nanopattern-modified SF electrospun scaffold presented satisfactory biocompatibility. Moreover, the nanopattern modification could effectively regulate the corresponding vascularization and ectopic osteogenic abilities *in vivo*. Further comparison illustrated that 10 min-etched nanopatterns could significantly enhance the ectopic osteogenesis of SF electrospun scaffold, thus showing great application potential in the field of bone tissue engineering. In addition, this kind of non-woven porous structured SF scaffold has also been widely used in the field of bladder [78], skin [79], nerve [80] and liver tissue engineering [81]. Therefore, related research could also provide valuable modification references for the development of other kinds of tissue engineering scaffolds.

## Conclusion

Effective nanopattern modification (nano-island patterns) on the 3D surface of the SF electrospun scaffold has been successfully achieved with air plasma etching. The features of related nanopatterns could be easily and effectively regulated by adjusting the corresponding etching time. This kind of surface modification strategy with low cost and convenience for different material forms and large-scale production properties showed great biomedical application potential. Appropriate nanopattern modification could effectively promote the cytocompatibility and specifical differentiation induction capacity. Compared with NE scaffold, 10E scaffold (10 min-etched) significantly enhanced stem cell adhesion and proliferation. However, as the etching time was further extended, the surface of 30E scaffold and 50E scaffold became more hydrophilic, and the interspacing between nanopatterns significantly increased, thus restricting cell adhesion and proliferation when compared to the unmodified group. Moreover, both *in vitro* and *in vivo* evaluations showed that 10E scaffold also presented the optimal osteogenic induction capacity. As the etching time becomes longer, both tensile mechanical properties and osteogenic induction capacity would be decreased. Altogether, it is expected that 10E scaffold has excellent application potential in the field of bone tissue repair. This type of modification obviously enhanced the proliferation and osteogenesis of BMSCs, while still maintaining 90% mechanical properties of its original state. Related studies have provided a facile and effective modification strategy for the important SF-based biomaterials and confirmed that appropriate modification could significantly promote their required biological functions. Hence, it informed future research for the design and mass production of efficient SF-based biomaterials.

## Supplementary Material

rbae117_Supplementary_Data

## Data Availability

The data supporting this study’s findings are available from the corresponding author upon reasonable request.
