# Supplementary material for: A facile nanopattern modification of silk fibroin electrospun scaffold and the corresponding impact on cell proliferation and osteogenesis
Source: Regen Biomater. 2024 Oct 1;11:rbae117. doi: 10.1093/rb/rbae117 (PMC11580685; doi:10.1093/rb/rbae117)
Supplement: rbae117_Supplementary_Data [file rbae117_supplementary_data.docx]

Supporting Information

**A facile nanopattern modification of silk fibroin electrospun scaffold and corresponding impact on cell proliferation and osteogenesis**

Xiaojiao Liu^†^, Qinjun Ouyang^†^, Xiang Yao^*^, Yaopeng Zhang^*^

State Key Laboratory for Modification of Chemical Fibers and Polymer Materials, College of Materials Science and Engineering, Shanghai Engineering Research Center of Nano-Biomaterials and Regenerative Medicine, Donghua University, Shanghai, 201620, P. R. China

^†^These authors contributed equally to this work.

^*^Correspondence address. E-mail: yaoxiang@dhu.edu.cn (X.Y.); zyp@dhu.edu.cn (Y.Z.)


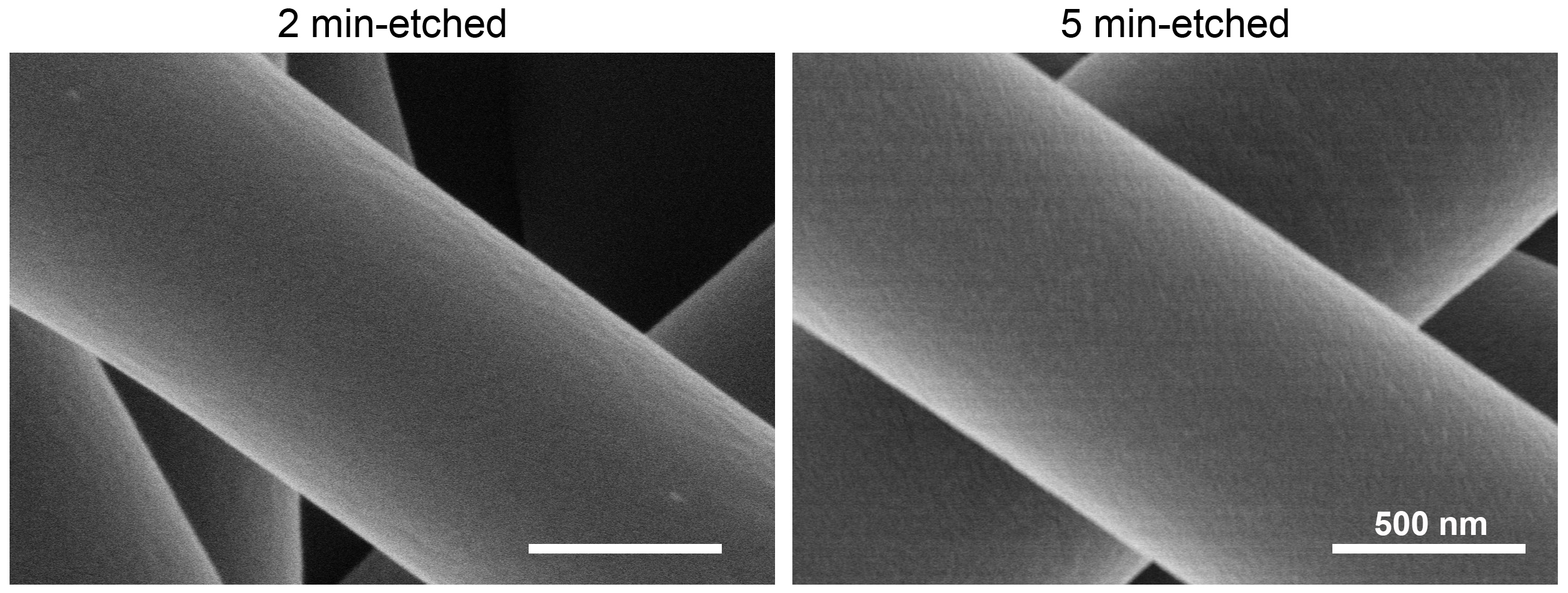


**Figure S1.** SEM images of the 2 min-etched and 5 min-etched SF electrospun scaffolds.


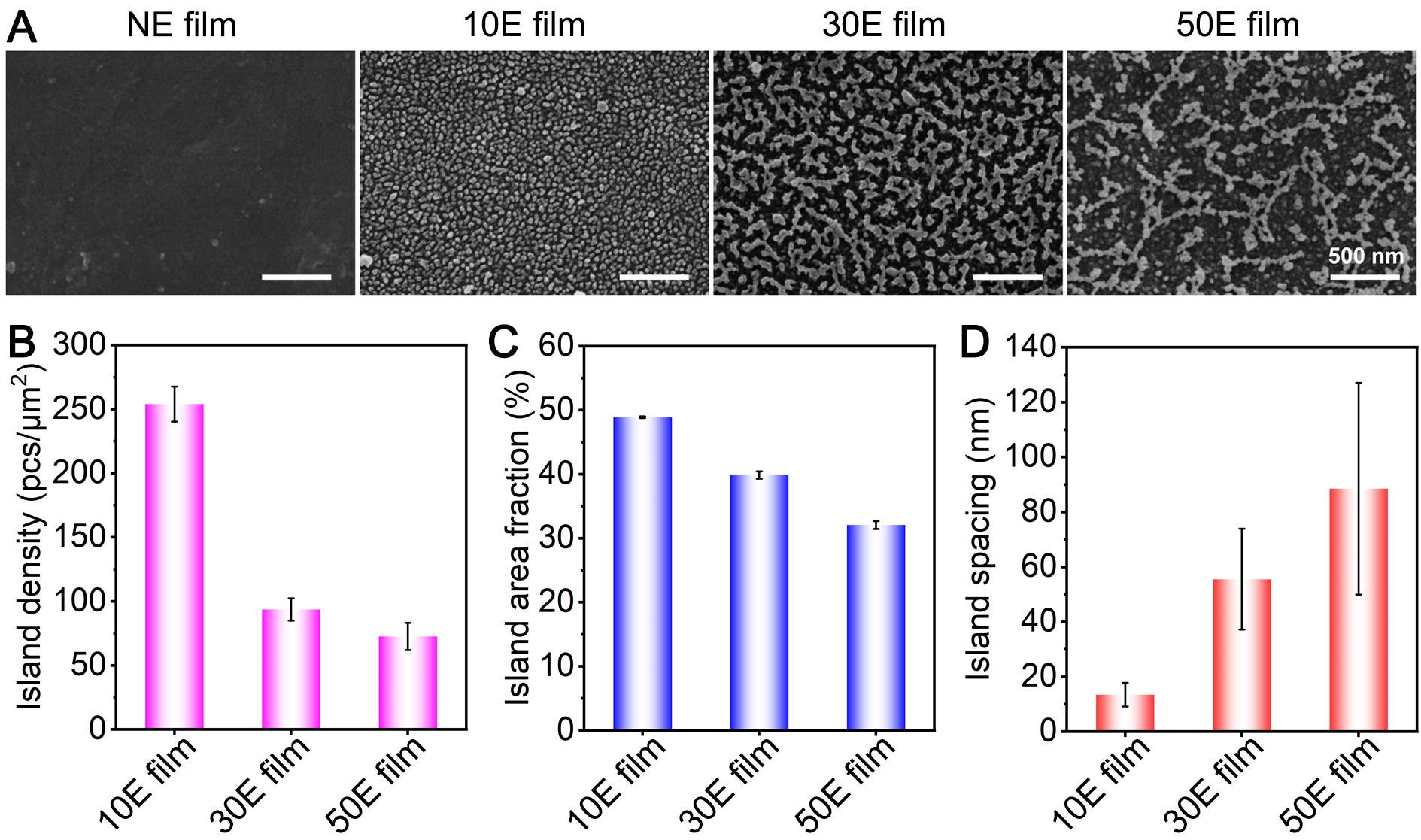


**Figure S2.** The morphologic and corresponding nanopatterned features of the non-etched or plasma-etched silk fibroin films with different etching times. (**A**) SEM images, (**B**) Island density, (**C**) Island area fraction, (**D**) Island spacing.


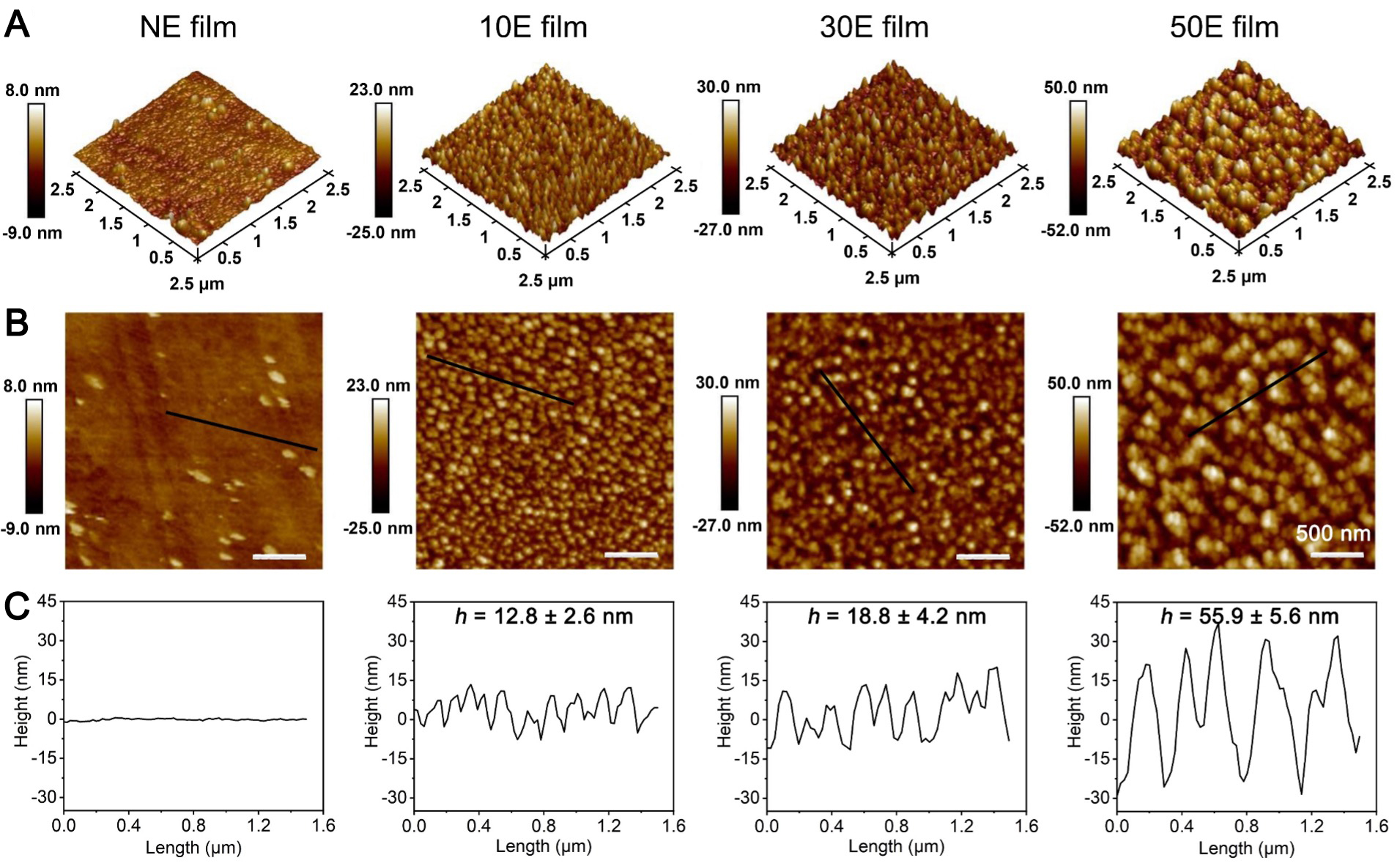


**Figure S3.** AFM characterization of the non-etched and plasma-etched silk fibroin films with different etching times. (**A**) 3D AFM images, (**B**) 2D AFM images, (**C**) Height information of the nanopatterns on the indicated lines of Figure S3B (2D AFM images).
